# Psychophysical Impact of COVID-19 Pandemic and Same-Sex Couples’ Conflict: The Mediating Effect of Internalized Sexual Stigma

**DOI:** 10.3389/fpsyg.2022.860260

**Published:** 2022-03-16

**Authors:** Jessica Pistella, Stefano Isolani, Salvatore Ioverno, Fiorenzo Laghi, Roberto Baiocco

**Affiliations:** ^1^Department of Developmental and Social Psychology, Sapienza University of Rome, Rome, Italy; ^2^Department of Sociology, Ghent University, Ghent, Belgium

**Keywords:** couples’ conflict, health, internalized sexual stigma, sexual minorities, COVID-19

## Abstract

Research on the effects of the COVID-19 pandemic on same-sex relationships is limited. The present study aimed at analyzing the association between the psychophysical impact of the COVID-19 pandemic and same-sex couples’ conflict, also considering the potential mediating effect of internalized sexual stigma (ISS). For this purpose, psychophysical challenges and couples’ conflict during the COVID-19 pandemic, ISS, age, biological sex, sexual orientation, relationship duration, religiosity, involvement in lesbian, gay, and bisexual (LGB) associations, sexual satisfaction, and interpersonal partner violence were assessed in an Italian sample of 232 LGB people engaged in a same-sex relationship (aged 18–45 years; *M*_*age*_ = 28.68, *SD* = 6.91). The results indicated that the psychophysical impact of the COVID-19 pandemic was significantly associated with couples’ conflict, and ISS mediated this relationship. Among the covariates considered, only sexual satisfaction was associated with couples’ conflict. The findings suggest that ISS, over and above the adverse effects of the COVID-19 pandemic on psychophysical health, triggered conflict within same-sex relationships. Studying the role of ISS in various relational and social contexts is important, as ISS may have an adverse effect on the mental health of sexual minority people. We recommend that more efforts be made to improve research on the LGB population during the public health response to the COVID-19 emergency, because the paucity of studies underlines the invisibility of this population in many domains, including the domain of romantic relationships. Implications and directions for future research are discussed.

## Introduction

The COVID-19 pandemic has seriously affected the mental health of the global population, and had a particularly negative impact on sexual minority people (Cahill et al., 2020; Salerno et al., 2020a,b) by, among other reasons, decreasing the quality of same-sex relationships (Li and Samp, 2021a). Previous research has shown that individuals involved in same-sex relationships are more likely to experience poor mental health and psychophysical harm during the COVID-19 pandemic compared to individuals in opposite-sex relationships (Li and Samp, 2021a), due to specific minority stressors (Meyer, 2003; Herek and McLemore, 2013). For instance, high levels of internalized sexual stigma (ISS), health disparities, reduced social support, social inequalities, and discrimination in accessing emergency government services have contributed to relationship dissatisfaction and conflict in this population during the emergency period (Gruberg, 2020; Salerno et al., 2020a; Li and Samp, 2021a). On this basis, drawing on a sample of lesbian, gay, and bisexual (LGB) Italian people, the present study aimed at: (a) examining the level of same-sex couples’ conflict during the Italian diffusion of COVID-19 and investigating the relationship between conflict and psychophysical impact of COVID-19 pandemic within these relationships; and (b) investigating the role of ISS—the most insidious subjective proximal minority stressor for sexual minority people—on the relationship between the psychophysical impact of the COVID-19 pandemic and same-sex couples’ conflict, after controlling for some individual (i.e., age, biological sex, sexual orientation) and contextual factors (i.e., religiosity, involvement in LGB associations, sexual satisfaction).

Studies focusing on same-sex couples’ conflict have shown that sexual minority people report comparable (Solomon et al., 2005) or lower (Gottman, 1994; Kurdek, 2004; Balsam et al., 2008) levels of conflict management compared to individuals in opposite-sex relationships. For instance, Solomon et al. (2005) found that married opposite-sex couples across the United States did not report more conflict about housework, money, or communication styles than same-sex couples (both female and male), despite discrepancies in the division of housework, finances, and relationship maintenance behaviors. The authors, in line with a previous study (LaSala, 2004), found that the area of conflict in which opposite-sex and same-sex couples most differed pertained to sex outside of the relationship, whereby sexual minority males were significantly more likely to experience conflict about non-monogamy than their male counterparts in opposite-sex marriages.

Another study in the United States (Balsam et al., 2008) found that same-sex couples reported more positive relationship quality and less conflict than opposite-sex couples; and within same-sex relationships, females reported fewer experiences of conflict relative to males. Again, Gottman (1994) underlined that relationship stability over time pertains to a couple’s ability to resolve conflict. Strategies for resolving conflict in a relationship include validating the partner’s feelings, reducing defensiveness, and adapting to the partner’s conflict style.

### Couples’ Conflict and Psychophysical Impact of COVID-19 Pandemic

Theoretical and empirical knowledge of the impact of the COVID-19 pandemic on same-sex relationships is limited (Li and Samp, 2021a). Thus, the following sections review the small body of existing literature on same-sex couples’ conflict associated with psychophysical challenges during the spread of COVID-19. Given the paucity of studies involving LGB couples, an overview of related research is presented, also considering studies on opposite-sex couples during the COVID-19 pandemic.

Some authors have analyzed the impact of the COVID-19 pandemic on adverse relationship processes (e.g., hostility, withdrawal, low responsive support) in opposite-sex (Günther-Bel et al., 2020; Luetke et al., 2020; Pietromonaco and Overall, 2021) and same-sex couples (Li and Samp, 2021a), studying the relevance of couples’ pre-existing individual and contextual factors (e.g., age, social class, minority status) that determine relationship quality. Quantitative and mixed-method studies with participants based in Spain (Günther-Bel et al., 2020) and the United States (Luetke et al., 2020) have demonstrated that the COVID-19 pandemic has led to significant relational conflict in opposite-sex couples. Li and Samp (2021a), studying sexual minority people in the United States, found that complaint avoidance mediated the relationship between the adverse impacts of the pandemic on daily life and relationship satisfaction, reducing positive conflict and crisis management between partners.

The COVID-19 pandemic forced some couples to reorganize their living situation, with some couples moving in together despite a lack of readiness for cohabiting (Fish et al., 2020; Singer, 2020). This dynamic may have exposed couples to potential relational difficulties due to the greater time spent together, a heightened vulnerability to disaccord, and the resurfacing of historical issues (Günther-Bel et al., 2020; Luetke et al., 2020; Li and Samp, 2021a). Such rapid cohabitation—or, conversely, the stress of separation—may have led some sexual minority individuals to come out to family members or significant others, despite a prior intention to remain private about their sexual orientation and romantic relationships (Fish et al., 2020; Li and Samp, 2021b). Consequently, same-sex partners may have experienced reduced social support due to their sexual minority status, and this may have increased conflict and tension within their romantic partnership (Archuleta et al., 2011; Keneski et al., 2018). In addition, both separation and confinement may have represented further stresses (Pietromonaco and Overall, 2021) that limited opportunities for positive conflict management, underlining the importance of prior relationship quality (Antonelli and Dèttore, 2014).

Thus, the COVID-19 pandemic has compromised the well-being of the general population (Luo et al., 2020; Shahyad and Mohammadi, 2020; Vindegaard and Benros, 2020; Zhu et al., 2020) and, in particular, the disadvantaged minority population (Gonzales et al., 2020; Salerno et al., 2020a; Li and Samp, 2021a), generating adverse psychophysical health consequences such as depression, anxiety, substance abuse (i.e., alcohol and cannabis abuse; Price, 2020), and cumulative psychological distress. For many same-sex couples, worry about COVID-19 and the daily life interruptions associated with the pandemic has significantly impacted relationship quality, satisfaction, and general well-being (Li and Samp, 2021a). Furthermore, in many cases, the stress caused by separation or confinement has disrupted the interaction between same-sex partners. As reported previously, rapid cohabitation or the stress of separation may have revealed non-heterosexual relationships to significant others, thereby increasing sexual minority peoples’ opportunities for experiencing rejection, adverse reactions, social exclusion, and discrimination.

### Minority Stress and the COVID-19 Pandemic

The theoretical framework of the minority stress model (Meyer, 1995, 2003), which holds that prejudice, vigilance, isolation, and discrimination are unique and chronic stressors among minority populations, may be applied to understand the impact of COVID-19 (and related factors) on LGB couples’ psychophysical health. Previous studies have shown that minority stress is associated with adverse effects on both physical (Diamant and Wold, 2003) and psychological health (D’Augelli et al., 1998; Cochran and Mays, 2006). Thus, the COVID-19 pandemic may represent an indirect mechanism through which same-sex couples experience distal (e.g., discrimination, violence, interpersonal homophobia) and proximal (e.g., ISS, fear of rejection) minority stress (Meyer, 1995, 2003), thereby exacerbating their existing relationships by reducing satisfaction and increasing conflict.

Research has defined ISS as the most insidious dimension of minority stress within the LGB population (Meyer, 2003). Specifically, ISS has been identified as a significant factor in romantic relationship satisfaction, conflict, and violence (Rollè et al., 2018; Sommantico et al., 2018; Li and Samp, 2021b). ISS is the product of society’s negative beliefs about sexual minority individuals, which some LGB people internalize; thus, it describes self-referred negative feelings and attitudes toward non-heterosexual sexual orientations (Mayfield, 2001; Herek and McLemore, 2013). Some researchers have found that LGB couples’ physical and psychological health is negatively associated with high levels of ISS (Sommantico et al., 2018). Other studies have reported that same-sex couples’ conflict and violence are correlated significantly and positively with ISS (Balsam and Szymanski, 2005; Carvalho et al., 2011; Rollè et al., 2018), highlighting the impact of this minority stressor on the quality of same-sex relationships. Previous research (Li and Samp, 2021a) has shown that complaint avoidance, conflict withholding, and higher levels of ISS damage same-sex relationships, leading to negative psychophysical consequences. Researchers have shown that contextual factors (e.g., worry about COVID-19; Pietromonaco and Overall, 2021) and minority stressors (e.g., ISS; Li and Samp, 2021a) may disrupt interactions between partners and increase conflict and discord.

### Variables Associated With (Same-Sex) Couples’ Conflict

Stanley et al. (2006) argued that, unless an analysis of same-sex couples’ conflict also considers individual and contextual factors, the representation of same-sex relationships will be incomplete, and possibly confused. Indeed, several individual characteristics may be associated with same-sex couples’ conflict and the adverse psychophysical challenges of sexual minority people during the COVID-19 pandemic, because pre-existing individual and contextual factors may predispose relationships to vulnerability. For instance, age, relationship duration, sexual satisfaction, and interpersonal partner violence [Intimate partner violence (IPV), defined as abusive behavior occurring within a romantic relationship, consisting of physical, sexual, or psychological violence] may significantly affect same-sex couples’ conflict and psychophysical impact of COVID-19 pandemic.

Luetke et al. (2020) showed that COVID-19–related relationship conflict in opposite-sex couples differed significantly by age group, with younger participants (age range of entire sample: 18–94 years) reporting higher levels of conflict. Regarding other socio-demographic variables, some studies have documented sexual orientation differences in couples’ conflict, whereby people self-identifying as bisexual report higher rates of conflict within same-sex relationships (Li and Samp, 2021a) and IPV (Whitfield et al., 2021) than lesbian women, gay men, and heterosexual persons. With respect to relationship duration, relationship stability over time has been shown to increase partners’ ability to resolve conflict through constructive and consolidated management (Gottman, 1994).

Although previous research has reported no significant differences between same-sex couples and opposite-sex couples regarding conflict over religious beliefs and involvement (Solomon et al., 2005), the present study included religiosity as a covariate, given that the study was conducted in Italy, where the Catholic Church’s symbolic power plays a decisive role in the lives of many sexual minority people (Baiocco and Pistella, 2019). In addition, lack of involvement in LGB associations has been linked to deficits in the constructive management of conflict and increased psychophysical problems. Lorenzi et al. (2015), in line with previous research (Russell and Richards, 2003), suggested that LGB associations constitute a significant source of social support for same-sex couples, enhancing their positive coping strategies, bestowing essential skills, and helping LGB people to become more capable of resolving conflict in their romantic relationships. Studies have also demonstrated that sexual satisfaction in same-sex and opposite-sex couples (Gottman et al., 2003; Cahill et al., 2020; Mendoza et al., 2020) is a protective factor for couples’ well-being. Moreover, sexual satisfaction is a relevant index of life quality, associated with psychophysical problems (Fleishman et al., 2020).

Finally, although previous research has shown that same-sex couples report comparable or lower levels of conflict management compared to opposite-sex couples (Gottman, 1994; Kurdek, 2004; Solomon et al., 2005; Balsam et al., 2008), studies focused on IPV have highlighted that the phenomenon occurs in same-sex couples at a comparable (Rollè et al., 2018) or even higher rate (Graham et al., 2019; Whitfield et al., 2021) than it does in opposite-sex couples. Breiding et al. (2013) reported that over 50% of gay men and approximately 75% of lesbian women in their study were victims of psychological IPV; on this basis, they identified more than 4 million sexual minority people with an experience of IPV in the United States.

## The Present Study

Evidence suggests that psychophysical challenges may influence the degree of conflict within romantic relationships (Solomon et al., 2005; Gonzales et al., 2020; Li and Samp, 2021a,b). For example, Ogolsky and Gray (2016) showed that daily negative emotions mediated the relationship between conflict and reports of a partner’s relationship maintenance in a sample of same-sex couples in the United States. Other studies have highlighted that the adverse effects on psychophysical health of the COVID-19 pandemic (Pietromonaco and Overall, 2021) and higher levels of ISS (Li and Samp, 2021a) may predict couples’ conflict.

To our knowledge, very little research has investigated the relationship between the psychophysical impact of the COVID-19 pandemic and same-sex couples’ conflict, as well as the potential mediators of this relationship (Li and Samp, 2021a). We hypothesized a mediation model whereby ISS explained the association between the psychophysical impact of the COVID-19 pandemic and same-sex couples’ conflict, and we applied this model to a sample of Italian LGB people. Italy was deemed a good case study for this investigation involving ISS, as the country is defined by conservative and religious values (Lingiardi et al., 2016; Baiocco et al., 2019). In Italy, sexual stigma and negative attitudes are still widespread, and few supportive policies for sexual minority people have been enacted relative to other Western nations (Hässler et al., 2021).

In addition, the diffusion of COVID-19 in Italy and the consequent health emergency gave rise to numerous social distancing measures. For instance, from March 9th to June 3rd, 2020, Italy went into lockdown, depriving all residents of positive social relations with friends, significant others, and supportive figures. In particular, the elimination of LGB social events limited opportunities for LGB people to receive support from the LGB community. In addition, the restrictive measures forced many LGB people to move into homes that were potentially unsafe (e.g., family homes with unsupportive parents).

During the COVID-19 pandemic, many LGB people were at increased risk of experiencing family rejection, harassment, victimization, and associated negative psychophysical health consequences (Salerno et al., 2020a,b). Many sexual minority people who were not out were prevented from living their lives authentically with their same-sex partner; they thereby suffered from increased ISS and the fear of discovery, as well as the potential negative consequences of such discovery, including psychological/physical abuse or homelessness. In many cases, the risk of being outed also rose for LGB couples who moved in together, putting them at greater risk of experiencing, for example, negative reactions from unsupportive parents (Fish et al., 2020; Li and Samp, 2021b).

The present study involved a mediation model focused on ISS because this minority stressor is considered the most dangerous stressor in the model proposed by Meyer (2003), reflecting internalized negative attitudes toward the self with respect to one’s non-heterosexual sexual orientation (Baiocco and Pistella, 2019). To complement previous empirical investigations on this subject, the study aimed at examining the effect of the psychophysical impact of COVID-19 pandemic on same-sex couples’ conflict, both directly and indirectly (*via* ISS), also considering some individual and contextual factors as covariates.

Specifically, based on previous research, we hypothesized that: (1) The psychophysical impact of COVID-19 pandemic would be associated with higher levels of same-sex couples’ conflict (Hypothesis 1); (2) ISS would be related to the psychophysical impact of COVID-19 pandemic and increased levels of same-sex couples’ conflict (Hypothesis 2); and (3) participants’ ISS would mediate the association between the psychophysical impact of COVID-19 pandemic and same-sex couples’ conflict (Hypothesis 3). In addition, given that some socio-demographic characteristics (e.g., age, biological sex, sexual orientation, relationship duration, religiosity, involvement in LGB associations, sexual satisfaction, IPV) have been shown to be relevant predictors of couples’ conflict (Solomon et al., 2005; Riggle et al., 2014; Lorenzi et al., 2015; Cahill et al., 2020; Fleishman et al., 2020; Whitfield et al., 2021), these factors were included as covariates in the analyses.

## Materials and Methods

### Procedures

An Internet-based survey (requiring 15–20 min to complete) was administered using Qualtrics. To recruit participants, we contacted LGB associations and requested them to invite their members to contribute to the study. Most participants (63%) were recruited from LGB associations and organizations in Rome (Italy). The remaining 37% were contacted *via* professional mailing lists, advertisements posted on websites and social networks, and an online link to the survey. We clarified to participants that the purpose of the study was to investigate the quality of same-sex relationships in sexual minority people. This explanation was made intentionally generic because we did not want respondents to know the true research objectives. Participants were recruited online between October 2020 and February 2021.

The inclusion criteria to participate were: (a) Italian nationality; (b) lesbian, gay, or bisexual sexual orientation; (c) cisgender identity; (d) aged 18 years or older; and (e) in a same-sex romantic relationship for at least 5 months (Kılıç and Altınok, 2021). Based on these criteria, four participants were excluded because they were not Italian, seven were excluded because they were not cisgender or LGB, and two were excluded because they did not complete the entire set of questionnaires. The research did not include persons with other non-heterosexual sexual orientations and non-cisgender people, because previous studies have reported that the factors that affect psychophysical health in these populations are significantly different from those that impact LGB people; furthermore, the relational dynamics of these populations have also been found to differ in numerous respects. Accordingly, future research should seek to investigate the relevance of couples’ conflict in these populations.

Participation in the study was voluntary and anonymous, and informed consent was acquired from all respondents. No compensation was provided. In total, 95% of the questionnaires were entirely filled in. Prior to data collection, the research protocol was approved by the Ethics Commission of the Department of Developmental and Social Psychology of the Sapienza University of Rome (Italy). All procedures performed with human participants were conducted in accordance with the ethical standards of the institutional and/or national research committee and the 1964 Helsinki Declaration and its later amendments.

### Participants

The study sample consisted of 232 Italian participants (56% male; *n* = 101) who self-identified as lesbian women (18%; *n* = 41), gay men (35%; *n* = 80), and bisexual people (47%; *n* = 111). Participants ranged in age from 18–45 years (*M*_*age*_ = 28.68, *SD* = 6.91). Twenty-seven respondents had been engaged in a stable relationship for less than 1 year (12%; *n* = 27), 43 had been in a stable relationship for 6–10 years (18%; *n* = 43), and 18 had been in a stable relationship for more than 10 years (8%; *n* = 18); the majority of participants had been involved in a stable same-sex relationship for 1–5 years (62%; *n* = 144). Approximately 12% (*n* = 29) of participants were legally married or civilly united. Nearly 45% (*n* = 104) cohabited with their same-sex partner during the pandemic and had continuously lived with their partner since that time. Fewer than half of the participants (36%; *n* = 84) were not involved in any LGB association. Table 1 presents the demographic data and descriptive statistics of the measures.

**TABLE 1:** Descriptive (means, standard deviations, percentages) of the sample’s characteristics.

	Females (*n* = 131) *M*(SD) or *n*(%)	Males (*n* = 101) *M*(SD) or *n*(%)	Total sample (*n* = 232) *M*(SD) or *n*(%)	*t/F/*χ^2^	*p*
1. Couple’s conflict	2.35 (0.78)	2.24 (0.67)	2.30 (0.74)	1.03	0.31
2. Psychophysical impact of the COVID-19 pandemic	5.01 (1.14)	5.06 (1.08)	5.03 (1.11)	0.09	0.76
3. ISS	1.52 (0.56)	1.66 (0.58)	1.58 (0.57)	3.50	0.06
4. Age	26.83 (5.86)	31.07 (7.44)	28.68 (6.91)	–4.71	<0.001
5. Sexual orientation (lesbian/gay)	41 (31%)	80 (79%)	121 (52%)	52.46	<0.001
6. Relationship duration (<1 year)	18 (14%)	9 (9%)	27 (12%)	9.10	0.03
1–5 years	87 (66%)	57 (56%)	144 (62%)		
6–10 years	21 (16%)	22 (22%)	43 (18%)		
More than 10 years	5 (4%)	13 (13%)	18 (8%)		
7. Religiosity	1.48 (0.72)	1.44 (0.64)	1.46 (0.69)	0.50	0.61
8. LGB Associations (yes)	90 (69%)	58 (57%)	148 (64%)	3.14	0.07
9. Sexual satisfaction	5.75 (1.03)	5.52 (1.22)	5.66 (1.12)	2.43	0.12
10. IPV perpetrators	0.27 (0.40)	0.28 (0.33)	0.28 (0.36)	0.02	0.89
11. IPV victims	0.28 (0.39)	0.26 (0.33)	0.27 (0.37)	0.11	0.74

*ISS, internalized sexual stigma; IPV, interpersonal partner violence. The t/F/χ^2^ refers to the biological sex differences in the total sample (females and males). Standard deviations and percentages are in parentheses.*

### Measures

#### Sociodemographic Variables

Baseline sociodemographic variables included age, biological sex (0 = female; 1 = male), and relationship duration (0 = < 1 year; 1 = 1–5 years; 2 = 6–10 years; 3 = > 10 years). Religiosity was measured by asking participants to report their religious involvement on a 4-point Likert-type scale ranging from 1 (*low involvement*) to 4 (*high involvement*). Respondents were required to indicate whether they were involved in any LGB association at the time of study (0 = no; 1 = yes).

Participants were asked to indicate their sexual orientation using the following response alternatives: “gay,” “lesbian,” “bisexual,” or “other, please specify.” On this basis, a dichotomous variable was created (0 = lesbian/gay; 1 = bisexual). Participants who selected “other” self-identified as “queer” (*n* = 3), and were not included in the analysis. In addition, participants were asked to report their gender identity, as follows: 0 = woman; 1 = man; 2 = transgender, male to female; 3 = transgender, female to male; 4 = transgender, gender non-conforming; and 5 = other, please indicate. Participants who self-identified as transgender (*n* = 4) were not included in the analysis. Thus, given that we included all participants who self-identified as cisgender (i.e., whose birth-assigned sex and gender identity were aligned), we did not incorporate gender identity as a variable in our analyses.

#### Couples’ Conflict During the COVID-19 Pandemic

Inspired by previous research (Luetke et al., 2020), we used a 6-item measure to assess participants’ perception of conflict in their romantic relationship during the COVID-19 pandemic. Each item was rated on a 5-point Likert scale ranging from 1 (*strongly disagree*) to 5 (*strongly agree*), with higher scores indicating greater levels of conflict. An example item was: “Tension and conflict with my romantic partner increased during the spread of COVID-19.” In the present study, reliability analyses indicated a high degree of internal consistency, with Cronbach’s alpha = 0.79.

#### Psychophysical Impact of COVID-19 Pandemic

Participants were asked to indicate the adverse impacts of the pandemic on their psychophysical health. Using the Coronavirus Impacts Questionnaire (Conway et al., 2020), participants responded to 2-items indexing the negative impacts of the COVID-19 pandemic on physical (e.g., “How has the spread of COVID-19 affected your physical well-being?”) and psychological health (e.g., “How has the spread of COVID-19 affected your psychological well-being?”). Each item was rated on a 7-point Likert scale ranging from 1 (*it improved considerably*) to 7 (*it worsened considerably*), with higher scores indicating greater negative impacts of the pandemic on psychophysical health. The correlation between these two items was high, *r* = 0.65.

#### Measure of Internalized Sexual Stigma – Short Version

A short version of the Measure of Internalized Sexual Stigma (MISS) was used to evaluate LGB people’s ISS, through 6-items (Lingiardi et al., 2012; Pistella et al., 2020). Example items included: “I do not believe in love between LGB people” and “I would prefer to be heterosexual.” Each item was rated on a 5-point Likert scale ranging from 1 (*I disagree*) to 5 (*I agree*), with higher scores indicating greater ISS. Cronbach’s alpha was 0.65.

#### Sexual Satisfaction

A short version of the New Scale of Sexual Satisfaction (Zheng and Zheng, 2017) was used to measure sexual satisfaction. Using a 3-item version, participants were asked to rate their satisfaction with the quality of their sex life during the prior 12 months, their desire toward their partner, and their sexual attraction (e.g., personal satisfaction with “the sexual activity in your relationship”). Responses were indicated on a 7-point Likert scale ranging from 1 (*extremely dissatisfied*) to 7 (*extremely satisfied*), with higher scores reflecting greater satisfaction with one’s sex life. The scale had high internal consistency, with Cronbach’s alpha = 0.85.

#### Intimate Partner Violence

The Conflict Tactics Scale Short Form (Straus and Douglas, 2004) is an 18-item measure that is used to investigate particular tactics for managing conflict in romantic relationships: *physical assault* (e.g., “I pushed, shoved, or slapped my partner”), *psychological aggression* (e.g., “I insulted or swore or shouted or yelled at my partner”), *injury from assault* (e.g., “I had a sprain, bruise, or small cut, or felt pain the next day because of a fight with my partner”), and *sexual coercion* [e.g., “I insisted on sex when my partner did not want to or insisted on sex without a condom (but did not use physical force”)]. Respondents are asked to indicate how many times a particular behavior has occurred over the prior year. Items evaluate both IPV perpetration (e.g., “I punched or kicked or beat-up my partner”) and IPV victimization (e.g., “My partner punched or kicked or beat me up”).

Previous research has revealed that the total score of violence on this scale is a proxy for violence within a romantic relationship (Straus et al., 1996), in which all subdomains of violence are averaged. A total score is derived from the 8-point Likert scale, ranging from 0 (*never in the past year*) to 7 (*more than 20 times in the past year*), with higher scores indicating more violence. Given that the present study aimed at controlling for previous episodes of violence within a romantic relationship, rather than examining different forms of IPV, we used the total score for all analyses. Cronbach’s alpha was 0.60 (IPV perpetration) and 0.61 (IPV victimization), with a high correlation between these dimensions, *r* = 0.71.

### Data Analysis

We conducted the analyses using the Statistical Package for the Social Sciences (SPSS 25.0). Group differences (in terms of biological sex) were examined using a Chi-square test, univariate analyses of variance, and *t*-tests. Bivariate correlations (Pearson’s *r*, two-tailed) were calculated to explore associations between couples’ conflict, the psychophysical impact of COVID-19 pandemic, ISS, and the other study variables.

Moreover, a mediation model analysis was employed to test the direct and mediating effects of the psychophysical impact of COVID-19 pandemic and ISS on same-sex couples’ conflict (with age, biological sex, sexual orientation, relationship duration, religiosity, involvement in LGB associations, sexual satisfaction, and IPV as covariate variables). We also examined moderated mediation models to verify the effect of biological sex in our model (Pace and Zappulla, 2013; Pace et al., 2018). We used the Process SPSS macro (Hayes, 2013) to evaluate the statistical significance of the direct and mediating effects with bias-corrected bootstrapping (5,000 samples) and 95% confidence intervals (CI). The continuous variables were standardized to *z*-scores prior to analysis, and non-normal variables (such as IPV perpetrators and victims) were logarithmically transformed before the regression association hypotheses were tested.

## Results

### Biological Sex Differences and Correlations Among the Study Variables

Table 1 presents the descriptive statistics of the measures, differentiated by biological sex. No biological sex differences were found in couples’ conflict, the psychophysical impact of COVID-19 pandemic, ISS, sexual satisfaction, and IPV subdimensions (i.e., perpetrators, victims). A Chi-square test detected a significant difference between females and males in sexual orientation, χ^2^(1,231) = 52.46, *p* < 0.001, showing that females (69%) self-identified as bisexual more frequently than males (21%). This result is aligned with the findings of previous studies. Additionally, an examination of the standardized residuals, χ^2^(1,231) = 9.10, *p* = 0.03, revealed that relationship duration was longer (>11 years) among males (13%), relative to females (4%).

We performed bivariate correlation analyses to examine the relationship between couples’ conflict, psychophysical impact of COVID-19 pandemic, ISS, and the other study measures (see Table 2). The results showed a significant positive moderate correlation between same-sex couples’ conflict and psychophysical challenges during the Italian spread of COVID-19. Furthermore, couples’ conflict and psychophysical impact of the COVID-19 pandemic were positively associated with ISS. Of note, couples’ conflict was negatively associated with age and sexual satisfaction. Finally, both IPV perpetration and IPV victimization were significantly correlated with couples’ conflict and sexual satisfaction.

**TABLE 2:** Correlations between couples’ conflict, the psychophysical impact of the COVID-19 pandemic, ISS, and other variables included in the study.

	1	2	3	4	5	6	7	8	9	10
1. Couple’s conflict	1.00									
2. Psychophysical impact of the COVID-19 pandemic	0.18**	1.00								
3. ISS	0.20**	0.15*	1.00							
4. Age	−0.18**	–0.05	–0.12	1.00						
5. Relationship duration	–0.12	–0.05	–0.12	0.54**	1.00					
6. Religiosity	–0.07	−0.14*	0.10	0.09	0.04	1.00				
7. LGB Associations	0.09	0.17**	−0.14*	–0.07	0.01	–0.11	1.00			
8. Sexual satisfaction	−0.21**	–0.07	–0.03	−0.24**	−0.22**	–0.02	–0.06	1.00		
9. IPV perpetrators	0.25**	0.03	0.06	–0.03	0.07	–0.05	–0.01	−0.27**	1.00	
10. IPV victims	0.27**	0.11	0.04	−0.15*	0.03	–0.08	0.04	−0.24**	0.71**	1.00

**p < 0.01, *p < 0.05. ISS, internalized sexual stigma.

### Couples’ Conflict, Psychophysical Impact of COVID-19 Pandemic, and Internalized Sexual Stigma: A Mediation Model

**FIGURE 1:**
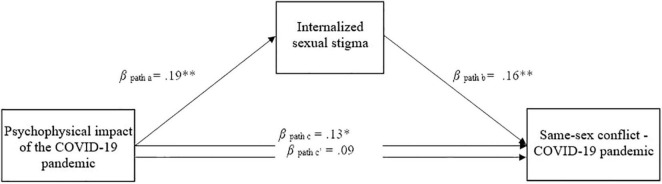
The mediated effect of internalized sexual stigma on the relationship between the psychophysical impact of the COVID-19 pandemic and couples conflict (*n* = 232). *p < 0.05, **p < 0.1. All values are beta coefficients.

We constructed a mediation model in which the relationship between self-perception of psychophysical challenges and couples’ conflict during the COVID-19 pandemic was mediated by the ISS of sexual minority participants. We adjusted the analyses for age, biological sex, sexual orientation (lesbian/gay vs. bisexual), relationship duration, religiosity, involvement in LGB associations, sexual satisfaction, and IPV perpetration and victimization. Figure 1 displays the results.

To test our first hypothesis, we ran our model without the mediator, and found a significant association between the psychophysical impact of the COVID-19 pandemic and couples’ conflict during the spread of COVID-19 (β path c in Figure 1). To test our second hypothesis, we entered ISS as a predictor of same-sex couples’ conflict. The results showed that ISS was significantly and positively associated with conflict levels. When we entered ISS as a mediator, the direct effect between the psychophysical impact of COVID-19 pandemic and couples’ conflict lost significance (β path c in Figure 1), providing support for our third hypothesis. The individual paths revealed that the psychophysical impact of the COVID-19 pandemic was positively related to ISS (β path a), which, in turn, was positively related to high levels of couples’ conflict (β path b). Psychophysical impact of COVID-19 pandemic and ISS accounted for a significant degree of variance in same-sex couples’ conflict, *F*(11, 220) = 4.77, *p* < 0.001, *R*^2^ = 0.20.

The indirect effects showed that ISS significantly mediated the association between psychophysical impact of COVID-19 pandemic and couples’ conflict [bootstrapping estimate = 0.04, *SE* = 0.02, 95% CI (0.01,0.07)]. Among the covariates considered in the model, only sexual satisfaction, β = −0.18, *SE* = 0.07, *p* < 0.01, was associated with couples’ conflict, while age, β = −0.08, *SE* = 0.08, *p* = 0.33; biological sex, β = −0.03, *SE* = 0.14, *p* = 0.86; sexual orientation, β = 0.18, *SE* = 0.15, *p* = 0.21; relationship duration, β = −0.09, *SE* = 0.07, *p* = 0.24; religiosity, β = −0.04, *SE* = 0.06, *p* = 0.57; involvement in LGB associations, β = 0.12, *SE* = 0.13, *p* = 0.36; IPV perpetration, β = 0.25, *SE* = 0.24, *p* = 0.28; and IPV victimization, β = 0.34, *SE* = 0.24, *p* = 0.16, were not.

In addition, we verified the effect of biological sex as a moderator in this relationship, but found no significant findings. Finally, given that sexual satisfaction was negatively related to couples’ conflict (*r* = −0.21, *p* < 0.01), an alternative model was tested using the same key variables, and ISS and sexual satisfaction as mediators (results available upon request). Specifically, we tested a mediation model in which couples’ conflict was the dependent variable, the psychophysical impact of the COVID-19 pandemic was the independent variable, and ISS and sexual satisfaction were the mediators. However, the association between the psychophysical impact of the COVID-19 pandemic and sexual satisfaction was not significant. Therefore, we evaluated our original model as the most adequate for describing the association between the psychophysical impact of the COVID-19 pandemic and couples’ conflict.

## Discussion

The present study aimed at extending existing knowledge about the relationship between psychophysical challenges and same-sex couples’ conflict during the COVID-19 pandemic, drawing on a sample of 232 LGB participants engaged in a same-sex relationship. In particular, the research contributed to our understanding of the relationship between the psychophysical impact of the COVID-19 pandemic and same-sex couples’ conflict, as well as our understanding of ISS as a potential mediator of this relation. While previous research (Solomon et al., 2005; Ogolsky and Gray, 2016; Gonzales et al., 2020) has suggested that psychophysical challenges may impact couples’ conflict, to our knowledge, only one study (Li and Samp, 2021a) has examined similar variables during the COVID-19 pandemic, including potential mediators that may explain their association.

In line with previous research, our analyses showed no significant differences between sexual minority females and sexual minority males with respect to ISS (Pistella et al., 2020), sexual satisfaction (Gottman et al., 2003), and IPV subdimensions (Balsam et al., 2008; Chong et al., 2013). Contrary to the literature, which shows that females report lower levels of couples’ conflict (Kurdek, 2004; Solomon et al., 2005) and are more likely to suffer from adverse psychophysical health during the pandemic period compared to males (Luo et al., 2020; Vindegaard and Benros, 2020), we found no differences between females and males on either of these two variables. It is likely that our failure to identify biological sex differences regarding couples’ conflict was due to the fact that we did not investigate all areas in which female and male couples have been found to differ, such as attitudes toward sex outside of the relationship (Solomon et al., 2005).

A possible explanation for the lack of biological sex differences with respect to the psychophysical impact of the COVID-19 pandemic is that previous studies have included a sample of opposite-sex couples, and research suggests that LGB people (and in fact all disadvantaged populations) develop skills for tolerating difficult emotions and coping with feared events (e.g., discrimination, violence, social isolation, rejection) in unsupportive and unsafe environments (Ryan et al., 2009; Antonelli and Dèttore, 2014). Such skills and coping strategies may have protected LGB people from the deleterious effects of the COVID-19 pandemic, regardless of their biological sex.

In line with our first hypothesis, we found a significant negative association between psychophysical challenges and same-sex couples’ conflict during the Italian spread of COVID-19. This finding is aligned with the results of prior research showing that daily negative feelings and adverse health outcomes may influence the degree of couples’ conflict (Ogolsky and Gray, 2016; Li and Samp, 2021a). Li and Samp (2021a) found a strong association between pandemic-related factors and individual well-being (e.g., anxiety, depression) among sexual minority people. The same authors reported that daily life interruptions during the pandemic significantly impacted same-sex couples’ conflict management.

Thus, we tested the association between ISS and same-sex couples’ conflict. The results supported our second hypothesis, suggesting that ISS was the primary variable predicting conflict in same-sex relationships. This finding is not surprising, given that higher ISS has been related to lower relationship quality in same-sex couples (Li and Samp, 2021b). The scale used to evaluate ISS in the present study contained the item “I do not believe in love between LGB people”; such an attitude may generate difficulties in conflict management for same-sex partners, especially with respect to relationship acceptance (Lingiardi et al., 2012). People with higher ISS often feel less confident about their same-sex relationships and are more likely to engage in hostile and conflictual conversations with their partners (Li and Samp, 2019).

In addition, ISS may imply the presence of a *don’t ask, don’t tell* attitude that prevents sexual minority people from being open about their sexual identity. Such an attitude is typical of the Italian context and may—both directly and indirectly—increase conflict within same-sex relationships (Fish et al., 2020; Pistella et al., 2020). Indeed, some sexual minority people avoid disclosing their sexual identity, for fear of adverse consequences, including negative reactions, isolation, and discrimination. As mentioned previously, the trend toward rapid cohabitation during the COVID-19 pandemic may have led many LGB people to come out to significant others, even if they may not have previously intended to disclose their romantic relationship. Added to this, the stress of being separated from one’s partner may have encouraged same-sex couples to come out to their families—despite the *don’t ask, don’t tell* culture—given that, if they were living at home during the pandemic, they may have been prevented from living out their romantic relationship. Thus, high ISS levels may have increased couples’ conflict due to a fear of disclosing their sexual identity. However, this explanation is only speculative, and is not supported by data from the present study.

The final model confirmed our third hypothesis about the mediating role of ISS in the association between psychophysical challenges and same-sex couples’ conflict during the COVID-19 emergency. Importantly, in the mediation models tested to verify the third hypothesis, ISS was found to mediate the relationship between the psychophysical impact of the COVID-19 pandemic and same-sex couples’ conflict in LGB participants. This finding has remarkable implications for our understanding of the underlying mechanism of increased conflict in same-sex relationships. Specifically, the mediation effect supports the explanation that ISS, over and above the adverse effects of the COVID-19 pandemic on psychophysical health, arouses conflict within same-sex couples, regardless of the partners’ age, biological sex, sexual orientation, relationship duration, religiosity, involvement in LGB associations, and history of IPV.

## Limitations and Future Directions

Although the present research has numerous strengths, several limitations should also be noted. First, the study was based on a convenience sample, and it was geographically restricted to Italy, thereby limiting the generalizability of the results. Another limitation regards the use of self-report measures, which may be influenced by social desirability. Furthermore, we recruited LGB people (Pistella et al., 2019), and our results may not apply to other sexual and gender minority individuals (e.g., queer, pansexual, and transgender people). Additionally, we did not consider some variables that are likely to relate to same-sex couples’ conflict, such as emotional and sexual infidelity, coming out, and political orientation.

Moreover, we did not analyze participants’ levels of positive LGB identity (Riggle et al., 2014; Petrocchi et al., 2020), even though this may significantly predict ISS and same-sex couples’ conflict, and represent a protective factor against stress resulting from the COVID-19 pandemic. Accordingly, this variable should be considered in future research. In the present study, the adverse effects of the pandemic on psychophysical health were determined *via* two items, which were not supported by standardized measures. Recently, a scale was developed for assessing the psychophysical impact of the COVID-19 pandemic (Coronavirus Impacts Questionnaire; Conway et al., 2020); however, this measure has not yet been validated, and its psychometric properties have not been tested.

Future research should consider the role of the perceived threat of COVID-19 retrospectively, in addition to other pandemic-related factors, such as a COVID-19 diagnosis or symptoms, loss caused by the COVID-19 pandemic, or financial challenges associated with COVID-19. Finally, a main limitation of the study is that data were collected from only one partner in each same-sex couple. Successful recruitment of both same-sex partners should be a goal of future research, despite this being a widely acknowledged research challenge.

## Conclusion

Understanding the effects of the COVID-19 pandemic on same-sex relationships is a complex objective. The present study extended knowledge about the impact of the health emergency on same-sex couples’ relational and personal well-being, showing that ISS may represent one of the main factors contributing to negative and conflictual interactions within same-sex romantic relationships (Salerno et al., 2020a). Studying the role of ISS in various relational and social contexts is important, due to its negative consequences on well-being, positive identity, and mental health in sexual minority people (Hart, 1995). Attention is needed at the structural level to reduce the risk of discrimination for LGB people in any setting. In this vein, anti-discriminatory campaigns and training programs about the relevance of inclusive practices for the well-being of LGB couples are recommended, as such programs may help LGB people (and their partners) decrease their levels of ISS.

It is worth noting that the *International Guidelines on Disaster Response* fail to consider the needs of LGB populations (Salerno et al., 2020a). The paucity of research on the LGB population during the COVID-19 pandemic speaks to the invisibility of LGB persons in the current public health response to the COVID-19 emergency. However, protecting the health of LGB individuals is pivotal, given their greater risk for developing psychophysical problems as a result of both minority and pandemic stress.

During this health emergency, positive and affirming social interactions with LGB individuals must be maintained *via* online instruments, such as video conferencing and social media, to mitigate the negative effects of the abovementioned stressors (Baiocco et al., 2021). Furthermore, public health stakeholders should disseminate provider information and parental resources to promote family acceptance of LGB persons’ identities (Phillips et al., 2020), and thereby improve the mental and physical health and well-being of sexual minority people. Finally, public agencies should make detailed and clear statements about the well-being of LGB people, increasing public awareness of the mental health vulnerabilities of LGB persons during the COVID-19 pandemic.

## Data Availability Statement

The raw data supporting the conclusions of this article will be made available by the authors, without undue reservation.

## Ethics Statement

The studies involving human participants were reviewed and approved by the Ethics Commission of the Department of Developmental and Social Psychology, Sapienza University of Rome. The patients/participants provided their written informed consent to participate in this study.

## Author Contributions

RB, FL, and SIs: conception and design. JP, SIo, and SIs: material preparation and data collection. RB, JP, and SIo: methodology, formal analyses, and investigation. JP and SIs: writing – original draft preparation. RB and FL: writing – review and editing. All authors read and approved the final manuscript.

## Conflict of Interest

The authors declare that the research was conducted in the absence of any commercial or financial relationships that could be construed as a potential conflict of interest.

## Publisher’s Note

All claims expressed in this article are solely those of the authors and do not necessarily represent those of their affiliated organizations, or those of the publisher, the editors and the reviewers. Any product that may be evaluated in this article, or claim that may be made by its manufacturer, is not guaranteed or endorsed by the publisher.

## References

[B1] Antonelli P. Dèttore D. (2014). Relationship, social, and individual well-being in Italian male same-sex couples. *J. Gay Lesb. Soc. Serv.* 26 383–406. 10.1111/famp.12078 24867576

[B2] Archuleta K. L. Britt S. L. Tonn T. J. Grable J. E. (2011). Financial satisfaction and financial stressors in marital satisfaction. *Psychol. Rep.* 108 563–576. 10.2466/07.21.PR0.108.2.563-576 21675570

[B3] Baiocco R. Pistella J. (2019). “Be as you are” clinical research center at the Sapienza University of Rome. *J. Gay Lesb. Mental Health* 23 376–379. 10.1080/19359705.2019.1644572

[B4] Baiocco R. Pezzella A. Pistella J. Papadopulous I. (2021). LGBT+ training needs for health and social care professionals: A cross-cultural comparison among seven European countries. *Sexual. Res. Soc. Policy* 19 22–36. 10.1007/s13178-020-00521-2

[B5] Baiocco R. Verrastro V. Fontanesi L. Ferrara M. P. Pistella J. (2019). The contributions of self-esteem, loneliness, and friendship to children’s happiness: The roles of gender and age. *Child Indicat. Res.* 12 1413–1433. 10.1007/s12187-018-9595-7

[B6] Balsam K. F. Szymanski D. M. (2005). Relationship quality and domestic violence in women’s same-sex relationships: The role of minority stress. *Psychol. Women Quart.* 29 258–269. 10.1111/j.1471-6402.2005.00220.x

[B7] Balsam K. F. Beauchaine T. P. Rothblum E. D. Solomon S. E. (2008). Three-year follow-up of same-sex couples who had civil unions in Vermont, same-sex couples not in civil unions, and heterosexual married couples. *Dev. Psychol.* 44 102–116. 10.1037/0012-1649.44.1.102 18194009

[B8] Breiding M. J. Chen J. Walters M. L. (2013). *The National Intimate Partner and Sexual Violence Survey (NISVS): 2010 Findings of Victimization by Sexual Orientation.* Atlanta: National center for injury prevention and control.

[B9] Cahill S. Grasso C. Keuroghlian A. Sciortino C. Mayer K. (2020). Sexual and gender minority health in the COVID-19 pandemic: Why data collection and combatting discrimination matter now more than ever. *Am. J. Public Health* 110 1360–1361. 10.2105/AJPH.2020.305829 32783729 PMC7427229

[B10] Carvalho A. F. Lewis R. J. Derlega V. J. Winstead B. A. Viggiano C. (2011). Internalized sexual minority stressors and same-sex intimate partner violence. *J. Fam. Viol.* 26 501–509. 10.1007/s10896-011-9384-2

[B11] Chong E. S. Mak W. W. Kwong M. M. (2013). Risk and protective factors of same-sex intimate partner violence in Hong Kong. *J. Interpers. Viol.* 28 1476–1497. 10.1177/0886260512468229 23295381

[B12] Cochran S. D. Mays V. M. (2006). “Estimating prevalence of mental and substance-using disorders among lesbians and gay men from existing national health data,” in *Sexual orientation and mental health: Examining identity and development in lesbian, gay, and bisexual people*, eds Omoto A. M. Kurtzman H. S. (Washington, D.C: American Psychological Association), 143–165. 10.1037/11261-007

[B13] Conway L. G. Woodard S. R. Zubrod A. (2020). *Social psychological measurements of COVID-19: Coronavirus perceived threat, government response, impacts, and experiences questionnaires* [Unpublished manuscript]. Montana: University of Montana. 10.31234/osf.io/z2x9a

[B14] D’Augelli A. R. Hershberger S. L. Pilkington N. W. (1998). Lesbian, gay, and bisexual youth and their families: Disclosure of sexual orientation and its consequences. *Am. J. Orthopsychiat.* 68 361–371. 10.1037/h0080345 9686289

[B15] Diamant A. L. Wold C. (2003). Sexual orientation and variation in physical and mental health status among women. *J. Women’s Health* 12 41–49. 10.1089/154099903321154130 12639368

[B16] Fish J. N. McInroy L. B. Paceley M. S. Williams N. D. Henderson S. Levine D. S. (2020). “I’m kinda stuck at home with unsupportive parents right now”: LGBTQ youths’ experiences with COVID-19 and the importance of online support. *J. Adolesc. Health* 67 450–452. 10.1016/j.jadohealth.2020.06.002 32591304 PMC7309741

[B17] Fleishman J. M. Crane B. Koch P. B. (2020). Correlates and predictors of sexual satisfaction for older adults in same-sex relationships. *J. Homosexual.* 67 1974–1998. 10.1080/00918369.2019.1618647 31172878

[B18] Gonzales G. de Mola E. L. Gavulic K. A. McKay T. Purcell C. (2020). Mental health needs among lesbian, gay, bisexual, and transgender college students during the COVID-19 pandemic. *J. Adolesc. Health* 67 645–648. 10.1016/j.jadohealth.2020.08.006 32933837 PMC10251764

[B19] Gottman J. M. (1994). *What predicts divorce? The relationship between marital processes and marital outcomes.* Mahwah NJ: Erlbaum.

[B20] Gottman J. M. Levenson R. W. Gross J. Frederickson B. L. McCoy K. Rosenthal L. (2003). Correlates of gay and lesbian couples’ relationship satisfaction and relationship dissolution. *J. Homosex.* 45 23–43. 10.1300/J082v45n01_02 14567652

[B21] Graham L. M. Jensen T. M. Givens A. D. Bowen G. L. Rizo C. F. (2019). Intimate partner violence among same-sex couples in college: A propensity score analysis. *J. Interpers. Viol.* 34 1583–1610. 10.1177/0886260516651628 27256495

[B22] Gruberg S. (2020). *An effective response to the coronavirus requires targeted assistance for LGBTQ people.* Washington, D.C: Center for American Progress.

[B23] Günther-Bel C. Vilaregut A. Carratala E. Torras-Garat S. P‘erez-Testor C. (2020). A mixed-method study of individual, couple, and parental functioning during the state-regulated COVID-19 lockdown in Spain. *Fam. Proc.* 59 1060–1079. 10.1111/famp.12585 32678461 PMC7405150

[B24] Hart J. (1995). Same Sex Couples and Counselling: The Development of a Multicultural Perspective. *J. Gay Lesb. Soc. Serv.* 3 89–108. 10.1300/J041v03n03_06

[B25] Hässler T. Ullrich J. Sebben S. Shnabel N. Bernardino M. Pistella J. (2021). Needs satisfaction in intergroup contact: A multi-national study of pathways toward social change. *J. Pers. Soc. Psychol.* 2021:si0000365. 10.1037/pspi0000365 34138605

[B26] Hayes A. F. (2013). *Introduction to mediation, moderation, and conditional process analysis: a regression-based approach.* New York, NY: Guilford.

[B27] Herek G. M. McLemore K. A. (2013). Sexual prejudice. *Annu. Rev. Psychol.* 64 309–333. 10.1146/annurev-psych-113011-143826 22994920

[B28] Keneski E. Neff L. A. Loving T. J. (2018). The importance of a few good friends: Perceived network support moderates the association between daily marital conflict and diurnal cortisol. *Soc. Psychol. Pers. Sci.* 9 962–971. 10.1177/1948550617731499

[B29] Kılıç N. Altınok A. (2021). Obsession and relationship satisfaction through the lens of jealousy and rumination. *Pers. Individ. Differ.* 179:110959. 10.1016/j.paid.2021.110959

[B30] Kurdek L. (2004). Are gay and lesbian cohabiting couples really different from heterosexual married couples? *J. Marriage Fam.* 66 880–900. 10.1111/j.0022-2445.2004.00060.x

[B31] LaSala M. C. (2004). Monogamy of the heart: Extradyadic sex and gay male couples. *J. Gay Lesb. Soc. Serv.* 17 1–24. 10.1300/J041v17n03_01

[B32] Li Y. Samp J. A. (2019). Internalized homophobia, language use, and relationship quality in same-sex romantic relationships. *Commun. Rep.* 32 15–28. 10.1080/08934215.2018.1545859

[B33] Li Y. Samp J. A. (2021a). The impact of the COVID-19 pandemic on same-sex couples’ conflict avoidance, relational quality, and mental health. *J. Soc. Pers. Relationsh.* 38 1819–1843. 10.1177/02654075211006199

[B34] Li Y. Samp J. A. (2021b). Antecedents to and outcomes of same-sex couples’ coming out talk. *Western J. Commun.* 85 1–21. 10.1080/10570314.2020.1748702

[B35] Lingiardi V. Baiocco R. Nardelli N. (2012). Measure of internalized sexual stigma for lesbians and gay men: a new scale. *J. Homosex.* 59 1191–1210. 10.1080/00918369.2012.712850 22966998

[B36] Lingiardi V. Nardelli N. Ioverno S. Falanga S. Di Chiacchio C. Tanzilli A. (2016). Homonegativity in Italy: Cultural issues, personality characteristics, and demographic correlates with negative attitudes toward lesbians and gay men. *Sexual. Res. Soc. Policy* 12 1–14. 10.1007/s13178-015-0197-6

[B37] Lorenzi G. Miscioscia M. Ronconi L. Pasquali C. E. Simonelli A. (2015). Internalized stigma and psychological well-being in gay men and lesbians in Italy and Belgium. *Soc. Sci.* 4 1229–1242. 10.3390/socsci4041229

[B38] Luetke M. Hensel D. Herbenick D. Rosenberg M. (2020). Romantic relationship conflict due to the COVID-19 pandemic and changes in intimate and sexual behaviors in a nationally representative sample of American adults. *J. Sex Marit. Therapy* 46 747–762. 10.1080/0092623X.2020.1810185 32878584

[B39] Luo M. Guo L. Yu M. Jiang W. Wang H. (2020). The psychological and mental impact of coronavirus disease 2019 (COVID-19) on medical staff and general public–A systematic review and meta-analysis. *Psychiat. Res.* 291:113190. 10.1016/j.psychres.2020.113190 32563745 PMC7276119

[B40] Mayfield W. (2001). The development of an internalized homonegativity inventory for gay men. *J. Homosex.* 41 53–76. 10.1300/J082v41n02_04 11482428

[B41] Mendoza N. S. Borders J. Sherman A. D. Thomas D. Harner V. Cimino A. (2020). Relationship commitment and alcohol use consequences among sexual minority women. *J. Gay Lesb. Soc. Serv.* 32 517–530. 10.1080/10538720.2020.1800546

[B42] Meyer I. H. (1995). Stress and mental health in gay men. *J. Health Soc. Behav.* 36 38–56. 10.2307/2137286 7738327

[B43] Meyer I. H. (2003). Prejudice, social stress, and mental health in lesbian, gay, and bisexual populations: Conceptual issues and research evidence. *Psychol. Bull.* 129 674–697. 10.1037/0033-2909.129.5.674 12956539 PMC2072932

[B44] Ogolsky B. G. Gray C. R. (2016). Conflict, negative emotion, and reports of partners’ relationship maintenance in same-sex couples. *J. Fam. Psychol.* 30 171–180. 10.1037/fam0000148 26322730

[B45] Pace U. Zappulla C. (2013). Detachment from parents, problem behaviors and the moderating role of parental support among Italian adolescents. *J. Fam. Issues* 6 768–783. 10.1177/0192513X12461908

[B46] Pace U. D’Urso G. Zappulla C. (2018). Adolescent effortful control as moderator of father’s psychological control in externalizing problems: A longitudinal study. *J. Psychol.* 153 164–177. 10.1080/00223980.2017.1419160 29405873

[B47] Petrocchi N. Pistella J. Salvati S. Carone N. Baiocco R. (2020). “I embrace my LGB identity”: The distinctive relevance of authenticity to well-being of Italian lesbians, gay men and bisexual people. *Sexual. Res. Soc. Policy* 17 75–86.

[B48] Phillips G. Felt D. Ruprecht M. M. Wang X. Xu J. Pérez-Bill E. (2020). Addressing the disproportionate impacts of the COVID-19 pandemic on sexual and gender minority populations in the United States: Actions toward equity. *LGBT Health* 7 279–282. 10.1089/lgbt.2020.0187 32790495 PMC8106250

[B49] Pietromonaco P. R. Overall N. C. (2021). Applying relationship science to evaluate how the COVID-19 pandemic may impact couples’ relationships. *Am. Psychol.* 76 438–450. 10.1037/amp0000714 32700937

[B50] Pistella J. Caricato V. Baiocco R. (2020). Coming out to siblings and parents in an Italian sample of lesbian women and gay men. *J. Child Fam. Stud.* 29 2916–2929. 10.1007/s10826-019-01597-0

[B51] Pistella J. Ioverno S. Russell S. T. (2019). The role of peer victimization, sexual identity, and gender on unhealthy weight control behaviors in a representative sample of Texas youth. *Int. J. Eating Disord.* 52 597–601. 10.1002/eat.23055 30805974 PMC10409614

[B52] Price A. (2020). Online gambling in the midst of COVID-19: A nexus of mental health concerns, substance use and financial stress. *Int. J. Mental Health Addic.* 20 362–379. 10.1007/s11469-020-00366-1 32837444 PMC7357671

[B53] Riggle E. D. Mohr J. J. Rostosky S. S. Fingerhut A. W. Balsam K. F. (2014). A multifactor Lesbian, Gay, and Bisexual Positive Identity Measure (LGB-PIM). *Psychol. Sexual Orientat. Gender Divers.* 1 398–411. 10.1037/sgd0000057

[B54] Rollè L. Giardina G. Caldarera A. M. Gerino E. Brustia P. (2018). When intimate partner violence meets same sex couples: A review of same sex intimate partner violence. *Front. Psychol.* 9:1506. 10.3389/fpsyg.2018.01506 30186202 PMC6113571

[B55] Russell G. M. Richards J. A. (2003). Stressor and resilience factors for lesbians, gay men, and bisexuals confronting antigay politics. *Am. J. Commun. Psychol.* 31 313–328. 10.1023/A:1023919022811 12866688

[B56] Ryan C. Huebner D. Diaz R. M. Sanchez J. (2009). Family rejection as a predictor of negative health outcomes in white and Latino lesbian, gay, and bisexual young adults. *Pediatrics* 123 346–352. 10.1542/peds.2007-3524 19117902

[B57] Salerno J. P. Devadas J. Pease M. Nketia B. Fish J. N. (2020a). Sexual and gender minority stress amid the COVID-19 pandemic: implications for LGBTQ young persons’ mental health and well-being. *Public Health Rep.* 135 721–727. 10.1177/0033354920954511 33026972 PMC7649999

[B58] Salerno J. P. Williams N. D. Gattamorta K. A. (2020b). LGBTQ populations: Psychologically vulnerable communities in the COVID-19 pandemic. *Psychol. Trauma Theory Res. Pract. Policy* 12 S239–S242. 10.1037/tra0000837 32551761 PMC8093609

[B59] Shahyad S. Mohammadi M. T. (2020). Psychological impacts of Covid-19 outbreak on mental health status of society individuals: a narrative review. *J. Military Med.* 22 184–192.

[B60] Singer J. (2020). *Are couples who moved in together for quarantine okay?.* Glamour. Available online at: https://www.glamour.com/story/are-couples-who-moved-in-together-for-quarantine-okay

[B61] Solomon S. E. Rothblum E. D. Balsam K. F. (2005). Money, housework, sex, and conflict: Same-sex couples in civil unions, those not in civil unions, and heterosexual married siblings. *Sex Roles* 52 561–575. 10.1007/s11199-005-3725-7

[B62] Sommantico M. De Rosa B. Parrello S. (2018). Internalized sexual stigma in Italian lesbians and gay men: The roles of outness, connectedness to the LGBT community, and relationship satisfaction. *J. Sex Marit. Therapy* 44 641–656. 10.1080/0092623X.2018.1447056 29494792

[B63] Stanley J. L. Bartholomew K. Taylor T. Oram D. Landolt M. (2006). Intimate violence in male same-sex relationships. *J. Fam. Viol.* 21 31–41. 10.1007/s10896-005-9008-9

[B64] Straus M. A. Douglas E. M. (2004). A short form of the Revised Conflict Tactics Scales, and typologies for severity and mutuality. *Viol. Victims* 19 507–520. 10.1891/vivi.19.5.507.63686 15844722

[B65] Straus M. A. Hamby S. L. Boney-McCoy S. Sugarman D. B. (1996). The revised conflict tactics scales (CTS2) development and preliminary psychometric data. *J. Fam. Issues* 17 283–316. 10.1177/019251396017003001

[B66] Vindegaard N. Benros M. E. (2020). COVID-19 pandemic and mental health consequences: Systematic review of the current evidence. *Brain Behav. Immun.* 89 531–542. 10.1016/j.bbi.2020.05.048 32485289 PMC7260522

[B67] Whitfield D. L. Coulter R. W. Langenderfer-Magruder L. Jacobson D. (2021). Experiences of intimate partner violence among lesbian, gay, bisexual, and transgender college students: The intersection of gender, race, and sexual orientation. *J. Interpers. Viol.* 36 11–12. 10.1177/0886260518812071 30453802 PMC8256635

[B68] Zheng L. Zheng Y. (2017). Sexual satisfaction in Chinese gay and bisexual men: Relationship to negative sexual minority identity and sexual role preference. *Sexual Relations. Therapy* 32 75–88. 10.1080/14681994.2016.1200027

[B69] Zhu S. Wu Y. Zhu C. Y. Hong W. C. Yu Z. X. Chen Z. K. (2020). The immediate mental health impacts of the COVID-19 pandemic among people with or without quarantine managements. *Brain Behav. Immun.* 87 56–58. 10.1016/j.bbi.2020.04.045 32315758 PMC7165285

